# Shox2 and Rassf1a DNA methylation: diagnostic utility and association with clinical stage, histological progression and gene mutational landscape in lung adenocarcinoma

**DOI:** 10.3389/fonc.2026.1727695

**Published:** 2026-01-28

**Authors:** Yixin Li, Yangli Zhang, Abida Alimu, Yulu Tan, Xiaojie Zhang, Linguo Xiang, Jia Li, Zhangling Liu

**Affiliations:** 1 The Center for Clinical Molecular Medical Detection, Innovative and Translational Laboratory of Molecular Diagnostics, Laboratory Medicine Center, The First Affiliated Hospital of Chongqing Medical University, Chongqing, China; 2 Biobank, The First Affiliated Hospital of Chongqing Medical University, Chongqing, China

**Keywords:** DNA methylation, driver mutations, LUAD, Rassf1a, Shox2

## Abstract

**Background:**

Lung cancer, characterized by its high global incidence and mortality rates, necessitates comprehensive and precise stratification strategies to guide the diverse diagnostic approaches and therapeutic agents in clinical decision-making.

**Objectives:**

Shox2 and Rassf1a promoter methylation are established biomarkers for the early screening of lung cancer. This study comprehensively investigated the clinical utility of Shox2 and Rassf1a promoter methylation alongside driver mutations for molecular subtyping and stratification in 1027 lung adenocarcinoma (LUAD) patients.

**Methods:**

This study included a cohort of 1027 LUAD patients who received treatment at the First Affiliated Hospital of Chongqing Medical University between January 2020 and August 2024. Comprehensive demographic and clinicopathological data were collected. Shox2 and Rassf1a methylation was quantified using the Lungme kit, while 10 driver mutations were detected by PCR assay. Chi-square tests were used to assess correlations between methylation status and clinicopathological characteristics; ROC analysis evaluated diagnostic performance for distinguishing LUAD subtypes. Multiple regression identified stage-associated hazardous factors.

**Results:**

In our cohort, Shox2 and Rassf1a methylation were correlated with more aggressive clinicopathological characteristics (age, sex, smoking, drinking, TNM stage and histological progression) and exhibited significant diagnostic potential for distinguishing early-stage lesions (adenocarcinoma *in situ* from LUAD across stages I-IV) and histological progression (minimally invasive adenocarcinoma versus invasive adenocarcinoma). Shox2 methylation exhibited significant co-occurrence with mutations of KRAS (*p* < 0.001) and MET (*p* = 0.02) and mutual exclusivity with mutations of EGFR (*p* < 0.001), RET (*p* = 0.013) and HER2 (*p* = 0.03). Rassf1a methylation showed no significant associations with these driver mutations. Combining Shox2 and Rassf1a methylation with EGFR mutations demonstrated enhanced discriminative capacity for early-stage lesions.

**Conclusions:**

Our study demonstrated that comprehensive analysis of methylation and gene mutations could provide a novel clinical strategy for molecular subtyping and precision medicine in LUAD.

## 1 Introduction

Lung cancer (LC) is the most prevalent cause of cancer-related death worldwide (1). Over the past decade, the widespread implementation of CT screening and the introduction of targeted therapies for lung cancer have significantly improved early diagnosis rates and survival outcomes for patients with advanced-stage disease. Paradoxically, the management of early-stage lung cancer remains challenged by both overtreatment and undertreatment risks stemming from inadequate prognostic biomarkers to identify patients who would benefit from timely pharmacologic interventions. Histopathological features were widely used to evaluate the invasiveness of LC. The histological progression of non-small cell lung adenocarcinoma (LUAD), the most common type of lung cancer, goes through the process of adenocarcinoma *in situ* (AIS) to minimally invasive adenocarcinoma (MIA) to invasive adenocarcinoma (IA). However, accurate pathological judgment requires complete resection of the nodule and proficiency of pathologist. Therefore, standard molecular prognostic stratification independent of complete resection surgery is imperative to avoid both overtreatment and missed opportunities in LUAD, especially in stage I.

Currently, molecular nucleic acid testing has been extensively integrated into clinical practice. In LUAD, DNA methylation analysis and genetic mutation detection have been used to guide targeted therapy selection and facilitate discrimination between malignant and benign pulmonary lesions. Beyond this, emerging studies indicate that aberrant DNA methylation levels and driver gene mutations significantly correlate with lung cancer prognosis, potentially offering complementary prognostic information. Short stature homeobox 2 (Shox2) functions as a growth-regulatory transcription factor that modulates cellular proliferation and differentiation programs. Notably, it orchestrates epithelial-mesenchymal transition, a pivotal process in oncogenic transformation (2); Ras-association domain family member 1 A (Rassf1a) functions as a canonical tumor suppressor that critically regulates cell cycle progression and metastasis-suppressing pathways (2). Their epigenetic silencing promotes oncogenic transformation across diverse malignancies. Aberrant hypermethylation of the promoters of Shox2 and Rassf1a manifest as a tumorigenesis-initiating event in LUAD. These two epigenetic markers now serve as clinically validated tools for early detection and diagnostic adjunct (3). It is reported that elevated methylation levels of Shox2 and Rassf1a exhibit significant associations with adverse clinical outcomes and advanced tumor grade (4–6). However, these preliminary findings require validation in larger cohorts due to limited sample sizes.

The advent of precision oncology has propelled the development of molecularly targeted agents against distinct driver gene mutations in LUAD. Currently, clinically approved targeted therapies encompass inhibitors for mutations of EGFR, ALK, KRAS, MET, ROS1, BRAF, RET, NTRK1/2/3, HER2, and PIK3CA. These oncogenic driver alterations exhibit significant prognostic implications in lung cancer, correlating with differential survival outcomes and therapeutic responses (7, 8). Given the high analytical sensitivity and reproducibility of nucleic acid detection technologies, integrative detection of genetic mutations and epigenetic methylation alterations may enable more refined and reliable prognostic stratification in lung cancer.

The objective of this study is to investigate the diagnostic efficacy of combined Shox2/Rassf1a methylation and driver genetic mutations in discriminating distinct invasive subtypes of lung adenocarcinoma, and to delineate their associations with clinicopathological characteristics. A total of 1027 cases were collected to analyze the distribution and frequency of methylated Shox2/Rassf1a and variants in 10 core driver genes. We explored the interaction between methylated Shox2/Rassf1a and genetic mutations, and the association of these DNA alterations with clinical characteristics. Lastly, we assessed the diagnostic performance of DNA methylation combined EGFR/KRAS mutations in discriminating lung adenocarcinoma subtypes (TNM grading and histological grading).

## 2 Materials and methods

### 2.1 Patients and specimens

**Figure 1:**
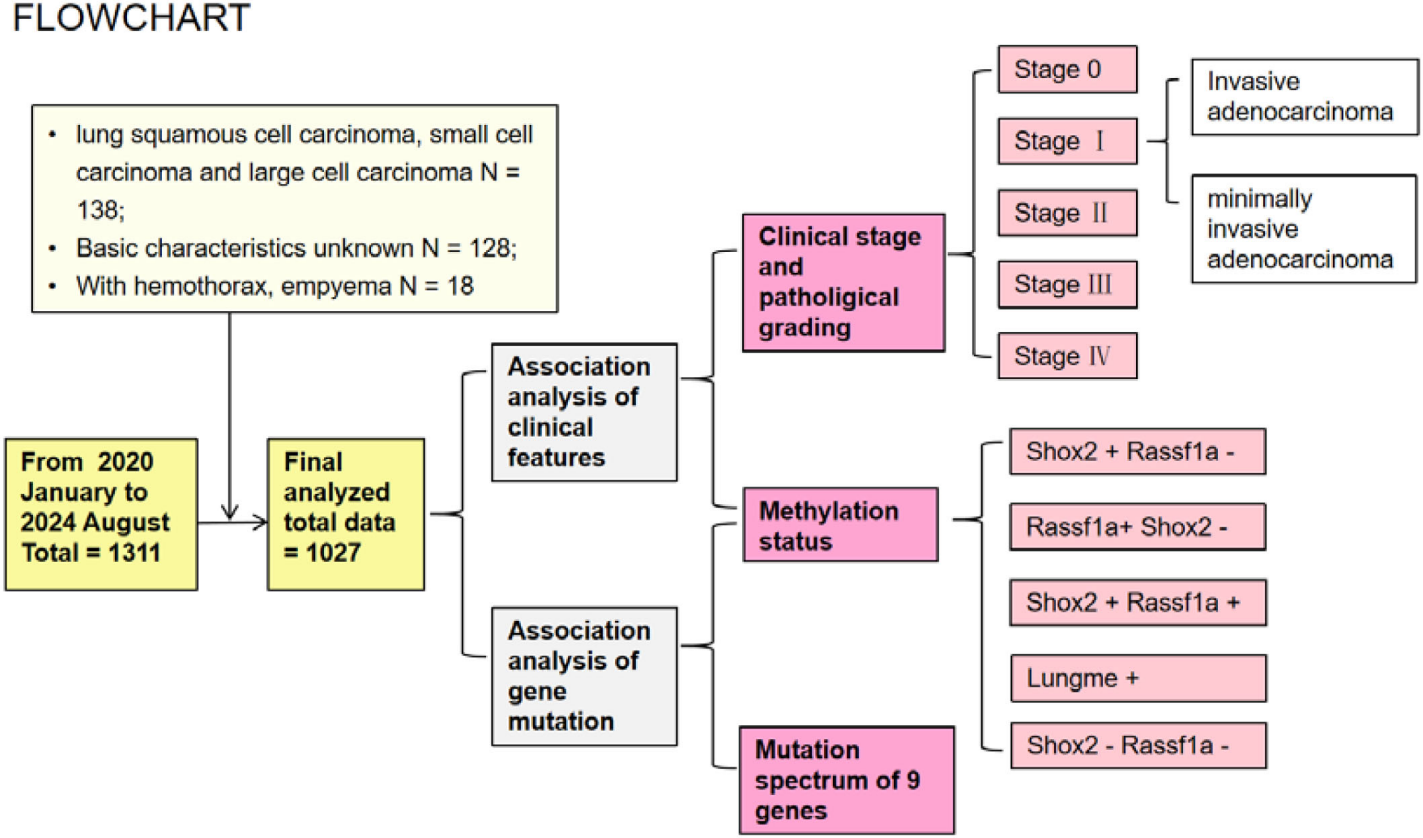
The flowchart.

This study was approved by the ethics committee of the First Affiliated Hospital of Chongqing Medical University with the registration number 2024-248-01. Only patients confirmed as lung adenocarcinoma (LUAD) by histomorphology were included. Patients with hemothorax, empyema or diagnosed as other types of lung cancer were excluded. A total of 1027 formalin-fixed paraffin-embedded (FFPE) specimens were collected from consenting individuals who were treated at the First Affiliated Hospital of Chongqing Medical University between January 2020 and August 2024. The flowchart has been provided in **Figure 1**. This study included 668 cases of invasive adenocarcinoma, 215 cases of minimally invasive adenocarcinoma, and 144 cases carcinoma *in situ*. The included patients comprised 402 men and 625 women with an average age of 59.0 years. The baseline characteristics of patients are shown in **Supplementary Table S1**.

### 2.2 Sample collection, molecular profiling, and pathological evaluation

Tissue samples consist of tumor tissues and cytological examinations. Cancer specimens were primarily collected from the Department of Thoracic Surgery and the Department of Respiratory Medicine. Driver gene mutation testing was required by clinicians based on the individual clinical status of each patient to guide targeted therapy decisions. The Center for Clinical Molecular Medical Detection at the First Affiliated Hospital of Chongqing Medical University conducted the detection and analysis to decide the driver gene mutation profiles. A database was settled up including information on patient’s age, gender, smoking history, family history, drinking, radiological features, pathological features and mutation types. The driver gene mutations analyzed include EGFR, KRAS, MET, BRAF, NTRK1/2/3, HER2, PIK3CA mutations, and *ALK*, *RET*, *ROS1* gene fusions.

The pathological diagnosis of NSCLC was classified according to the 2021WHO classification of Tumors of the Chest (5th Edition) (9). All pathological diagnoses were independently reviewed and confirmed by two experienced thoracic pathologists who were blinded to each other’s assessments and clinical data. Any diagnostic discrepancies were resolved by joint re-examination to reach a consensus, or by consultation with a third senior pathologist. In addition to the primary diagnosing pathologist, all specimens undergoing molecular testing required results review by two additional pathologists specialized in molecular test assessment, in accordance with departmental regulations. All cases were staged in accordance with the American Joint Committee on Cancer (AJCC) 8th edition TNM classification. The final TNM staging was determined by a multidisciplinary team (MDT) specializing in thoracic oncology, integrating radiologic, histopathologic, and clinical data. In cases with indeterminate findings, follow-up imaging or biopsy was conducted within 4 weeks to confirm the diagnosis.

### 2.3 DNA extraction and bisulfite treatment for methylation detection

The paraffin-embedded tissue was lysed using a DNA extraction kit (CWY009S, CW Biotech Co., Ltd., China) in accordance with the guidelines provided by the manufacturer. The Qubit dsDNA HS Assay Kit (Life Technologies Co., Carlsbad, CA) was employed to measure the concentrations of DNA on the Qubit Fluorometer 3.0. For each sample, 200 ng of DNA were subjected to sodium bisulfite treatment using the DNA Purification Kit (PF03X056, Tellgen Co., China).

### 2.4 Detection of Shox2 and Rassf1a methylation

The Lungme Real-time PCR Kit (Tellgen Co., Shanghai, China) was used to analyze the methylation level of Shox2 and Rassf1a as previously reported (10). The methylation-specific polymerase chain reaction (MS-PCR) process was conducted on the SLAN-96S platform (Hongshi Co., Shanghai, China) to amplify methylated Shox2, Rassf1a, and β-actin. The primers were as follows: Shox2 F: 5’- TTGTTTTTGGGTTCGGGTT-3’, R: 5’- CATAACGT AAACGCCTATACTC-3’; Rassf1a F: 5’- CGGGGTTC GTTTTGTGGTTTC-3’, R: 5’- CCGATTAAATCCGTAC TTCGC-3’.

### 2.5 Amplification refractory mutation system PCR (ARMS-PCR)

DNA and RNA were aspirated from FFPE or paraffin blocks using Nucleic Acid Extraction Reagent (cat. no.8.0223601X036G; Amoy Diagnostics Co., Ltd);, according to the manufacturer’s protocols. Prior to extraction, the paraffin blocks were sectioned into 4-μm thick sections according to standard procedures. The sections were stained with hematoxylin and eosin (H&E), and tumor cell content was assessed under microscopic evaluation. To ensure sufficient mutant allele detection, only samples containing ≥ 20% tumor cell nuclei were included. In cases with lower tumor cellularity, microdissection was performed to enrich the tumor cell fraction. DNA and RNA concentrations of all specimens were measured using a NanoDrop One spectrophotometer at 280 nm (Thermo Fisher Scientific, Inc.). The gene mutations of all samples were tested by amplification refractory mutation system-polymerase chain reaction (ARMS-PCR) and the thermo-cycling conditions of PCR were as follows: 1 cycle of 95°C for 5 min; followed by 10 cycles of 95°C for 25 s, 64°C for 20 s, 72°C for 20 s; and lastly 36 cycles of 93°C for 25 s, 60°C for 35 s, 72°C for 20 s. The reagents of ARMS-PCR were acquired from Amoy Dx Diagnostics Co., Ltd. (cat. no. 20143402001).

### 2.6 Receiver Operating Characteristic (ROC) analysis

ROC curves were used to assess the diagnostic effects of Shox2 and Rassf1a. Firstly, the △Ct values of Shox2 and Rassf1a methylation were used as predictive variables while minimally invasive adenocarcinoma (MIA) and invasive adenocarcinoma (IA) were used as outcome variables to create the ROC curve in stage I LUAD patients. Then, the ROC curves were constructed using the qualitative results of methylated Shox2 and methylated Rassf1a as predictive variables while adenocarcinoma *in situ* (AIS) and adenocarcinoma, MIA and IA as outcome variables. To assess the diagnostic effects of Shox2 and Rassf1a combined genetic mutations, the methylation of Shox2 and Rassf1a combined EGFR or KRAS mutations were used as predictive variables while adenocarcinoma *in situ* (AIS) and adenocarcinoma, MIA and IA as outcome variables.

### 2.7 Statistical analysis

The IBM SPSS Statistics (version 20.0) (SPSS Inc., Chicago, IL) and GraphPad Prism (version 9.0) software were used for statistical analysis and data visualization, respectively. The association of Shox2 and Rassf1a methylation with clinical characteristics, radiological features, clinical stage, pathological grade and mutations of 9 genes were analyzed by the chi-square test. The ROC curve was employed to assess the diagnostic efficacy of Shox2 and Rassf1a. Factors associated with clinical stage comparisons (IA vs. MIA and AIS vs. LA) were identified using logistic regression. For categorical variables, chi-square test or Fisher’s exact test was used as appropriate. A p-value of < 0.05 was considered statistically significant.

## 3 Results

### 3.1 Overview of study population

As shown in **Supplementary Table S1**, the proportion of female was higher than male in all clinical stages (0 - IV) of LUAD patients. Different clinical stages were significantly associated with smoking, radiological features and methylation status of Shox2 and Rassf1a. However, there were no significant correlations between clinical stages and drinking, family history and location of pulmonary nodules. The proportions of smoking were higher in stage I (194/722, 26.87%), II (23/78, 29.49%), III (20/65, 30.77%), IV (3/18, 16.67%) compared with stage 0 (17/144, 11.81%). We defined single-positive (Shox2+Rassf1a- or Shox2-Rassf1a+) or double-positive (Shox2+Rassf1a+) methylation status as lungme positive (lungme+). The entire positive rate of Shox2 and Rassf1a methylation (lungme+) was significantly higher in stage I (343/722, 47.51%), II (56/78, 71.79%), III (48/65, 73.85%), IV 9/18, 50%) than stage 0 (19/144, 13.19%).

### 3.2 Association of Shox2 and Rassf1a methylation with clinicopathological and radiological features in LUAD

**Figure 2:**
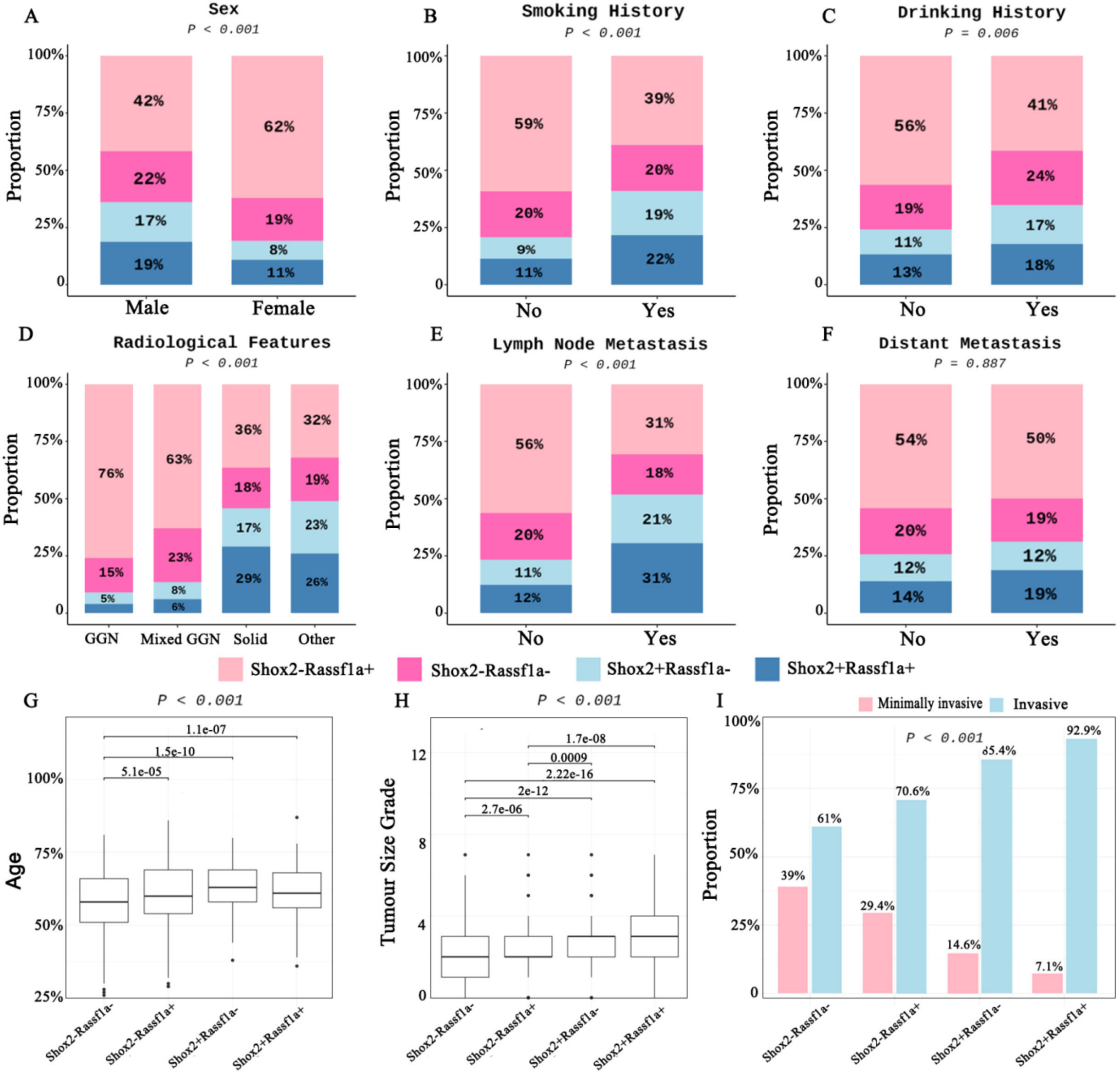
Analysis of Shox2 and Rassf1a methylation association with clinical characteristics and pathological diagnosis. **(A-F)** The correlation between Shox2 and Rassf1a methylation with sex, smoking history, drinking history, radiological features, lymph node metastasis, distant metastasis. **(G-I)** The correlation between Shox2 and Rassf1a methylation with age, tumor size, pathological staging.

Shox2 and Rassf1a methylation was correlated with sex, smoking, drinking. The positive rates of Shox2 and Rassf1a methylation were significantly higher in male (233/402, 58%) and patients with smoking (157/257, 61%) and drinking (91/154, 59%) (**Figures 2A–C**). For radiological features, the positive rate of Shox2 and Rassf1a methylation (132/206,64%) and the proportion of Shox2+Rassf1a+ (60/206, 29%) were higher in patients with solid nodules (**Figure 2D**). In patients with lymph node metastasis, the positive rate (60/87, 69%) and the proportion of Shox2+Rassf1a+ (30/87, 31%) were significantly higher than those without lymph node metastasis (**Figure 2E**). However, there were no significant differences in Shox2 and Rassf1a methylation between patients with or without distant metastasis (**Figure 2F**). Moreover, we analyzed age distribution and tumor size grade in different methylation status. Patients with Shox2 and/or Rassf1a methylation exhibited a significantly higher mean age and larger average tumor size than those without such methylation changes (**Figures 2G, H**). The proportion of IA was highest in the Shox2+Rassf1a+ group and lowest in the Shox2-Rassf1a- group, suggesting that Shox2 and Rassf1a methylation may be associated with greater tissue invasiveness. Furthermore, higher percentage of IA in Shox2+Rassf1a- group compared to Rassf1a+Shox2- group indicated that Shox2 methylation may be more strongly correlated with enhanced tissue invasiveness than Rassf1a methylation (**Figure 2I**).

### 3.3 Combined methylation analysis of Shox2 and Rassf1a enhances diagnostic accuracy for IA in stage I and LUAD

**Figure 3:**
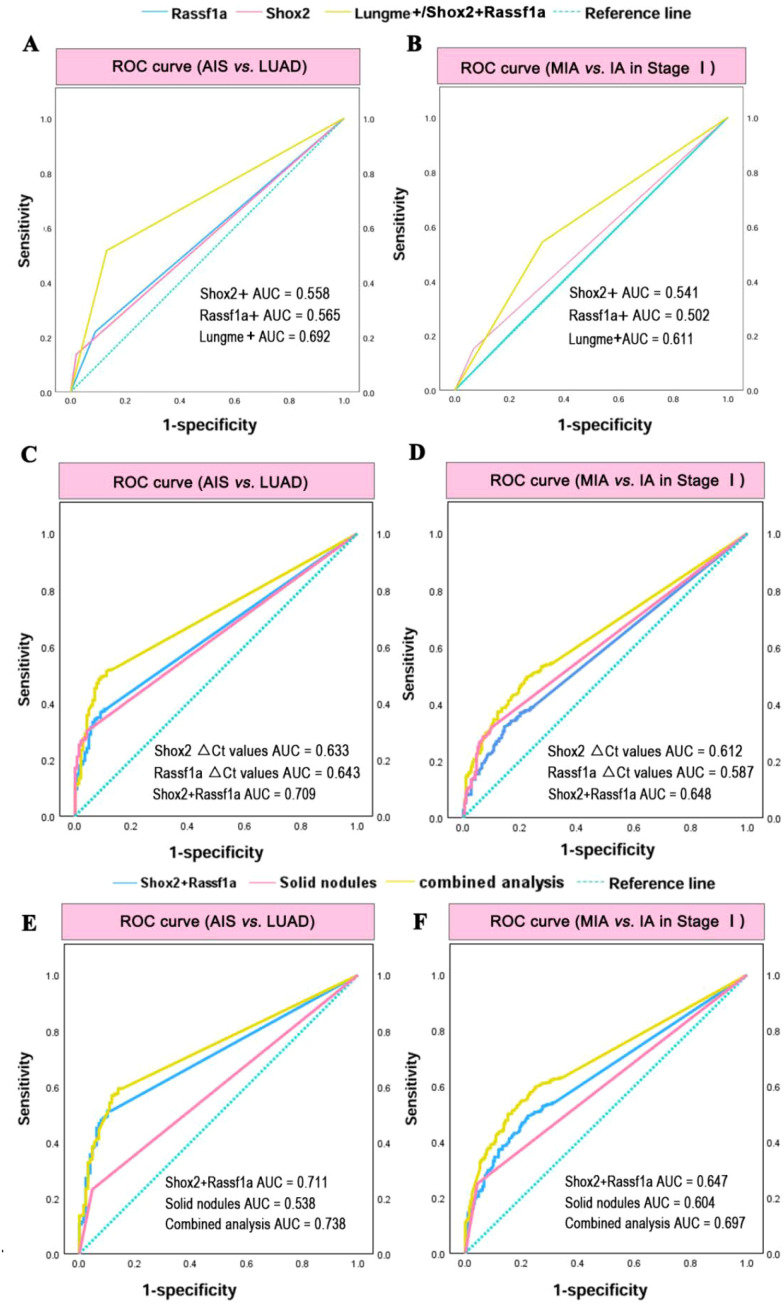
The diagnostic efficiency of Shox2 and Rassf1a methylation for IA and LUAD. **(A)** ROC analysis for discriminating AIS and LUAD (stage I-IV) based on Shox2 and Rassf1a methylation. **(B)** ROC analysis for discriminating MIA and IA in stage I based on Shox2 and Rassf1a methylation. **(C)** ROC analysis for discriminating AIS and LUAD based on Shxo2 and Rassf1a methylation △Ct Values. **(D)** ROC analysis for discriminating MIA and IA in stage I based on △Ct Values. **(E)** ROC analysis for discriminating AIS and LUAD combined solid nodules and methylation assays. **(F)** ROC analysis for discriminating MIA and IA in stage I combined solid nodules and methylation assays.

The relative levels of methylated Shox2 and Rassf1a were calculated as △Ct Shox2=Ct Shox2−Ct β-ACTB and △Ct Rassf1a = Ct Rassf1a – Ct β-ACTB. The △Ct values for negative methylation results (with undetectable Ct values) were set to 20. The diagnostic efficacies of Shox2 and Rassf1a methylation for AIS and LUAD were assessed through ROC curve analysis with the △Ct values. The AUC values for Shox2(△Ct) and Rassf1a(△Ct) were 0.633 and 0.643, respectively. The optimal cutoff value of Shox2(△Ct) for distinguishing AIS and LUAD was 15.5. With this cutoff, the corresponding sensitivity and specificity were 30.0% and 95.8%, respectively. The optimal cutoff value of Rassf1a(△Ct) was 11.1, and the corresponding sensitivity and specificity were 36.7% and 91.0%. The AUC value for the combined Shox2 and Rassf1a was 0.709, and the corresponding sensitivity and specificity were 51.3% and 88.9% (**Figure 3C**). Then, the ROC curve analysis was performed with the qualitative results of methylation. When applying its predefined qualitative threshold of the Lungme assay kit, the AUC values of individual Shox2, individual Rassf1a and combined analysis (Lungme+) were 0.558, 0.565 and 0.692 (**Figure 3A**). The Shox2 methylation positivity showed a sensitivity of 13.7% and a specificity of 97.9% in distinguishing AIS and LUAD. The Rassf1a methylation positivity showed a sensitivity of 21.9% and a specificity of 90.9%. The Lungme positivity demonstrated a sensitivity of 51.6% and a specificity of 86.8%.

Subsequently, diagnostic efficacies of Shox2 and Rassf1a methylation for MIA and IA in stage I were assessed through ROC curve analysis. The AUC values for Shox2(△Ct) and Rassf1a(△Ct) were 0.612 and 0.587, and the AUC value for combined Shox2 and Rassf1a was 0.648 (**Figure 3D**). The optimal cutoff value of △CtShox2 for distinguishing between IA and MIA was 15.5. With this cutoff, the corresponding sensitivity and specificity were 31.6% and 90.2%, respectively. The optimal cutoff value of △CtRassf1a was 7.7, the corresponding sensitivity and specificity were 32% and 85.6%. The optimized Lungme demonstrated a sensitivity of 49.5% and a specificity of 77.7%. When applying its predefined qualitative threshold of the Lungme assay kit, the AUC values of Shox2, Rassf1a and combined analysis (Lungme+) were 0.541, 0.502 and 0.611 (**Figure 3B**). The Shox2 methylation positivity showed a sensitivity of 15.2% and a specificity of 93.1% and the Rassf1a methylation positivity showed a sensitivity of 34.9% and a specificity of 77.3%. The Lungme positivity demonstrated a sensitivity of 54.2% and a specificity of 67.9%.

The description of solid nodules on imaging is associated with a higher likelihood of malignancy and greater aggressiveness. Therefore, we further analyzed the characteristics of solid nodules and evaluated the diagnostic performance of combining markers in differentiating the malignant potential of pulmonary nodules. For distinguishing between AIS and LUAD, the AUC values of Shox2+Rassf1a, Solid nodules and combined analysis were 0.711, 0.538, and 0.738, respectively (**Figure 3E**). For distinguishing between MIA and IA in stage I, the AUC values were 0.647, 0.604, and 0.697 (**Figure 3F**). These results demonstrated that combined methylation analysis of Shox2 and Rassf1a exhibited superior sensitivity and specificity compared with individual methylation analysis of either Shox2 or Rassf1a for the diagnosis of IA and LUAD. Optimizing the cutoff values can enhance the sensitivity and specificity of Shox2 and Rassf1a methylation assay in discriminating MIA and IA. Additionally, the methylation assay of Shox2 and Rassf1a could offer important supplementary information beyond imaging conclusions.

### 3.4 Methylation of Shox2 and Rassf1a and driver gene mutations: co-occurrence patterns and independent prognostic impact on IA and LUAD

**Figure 4:**
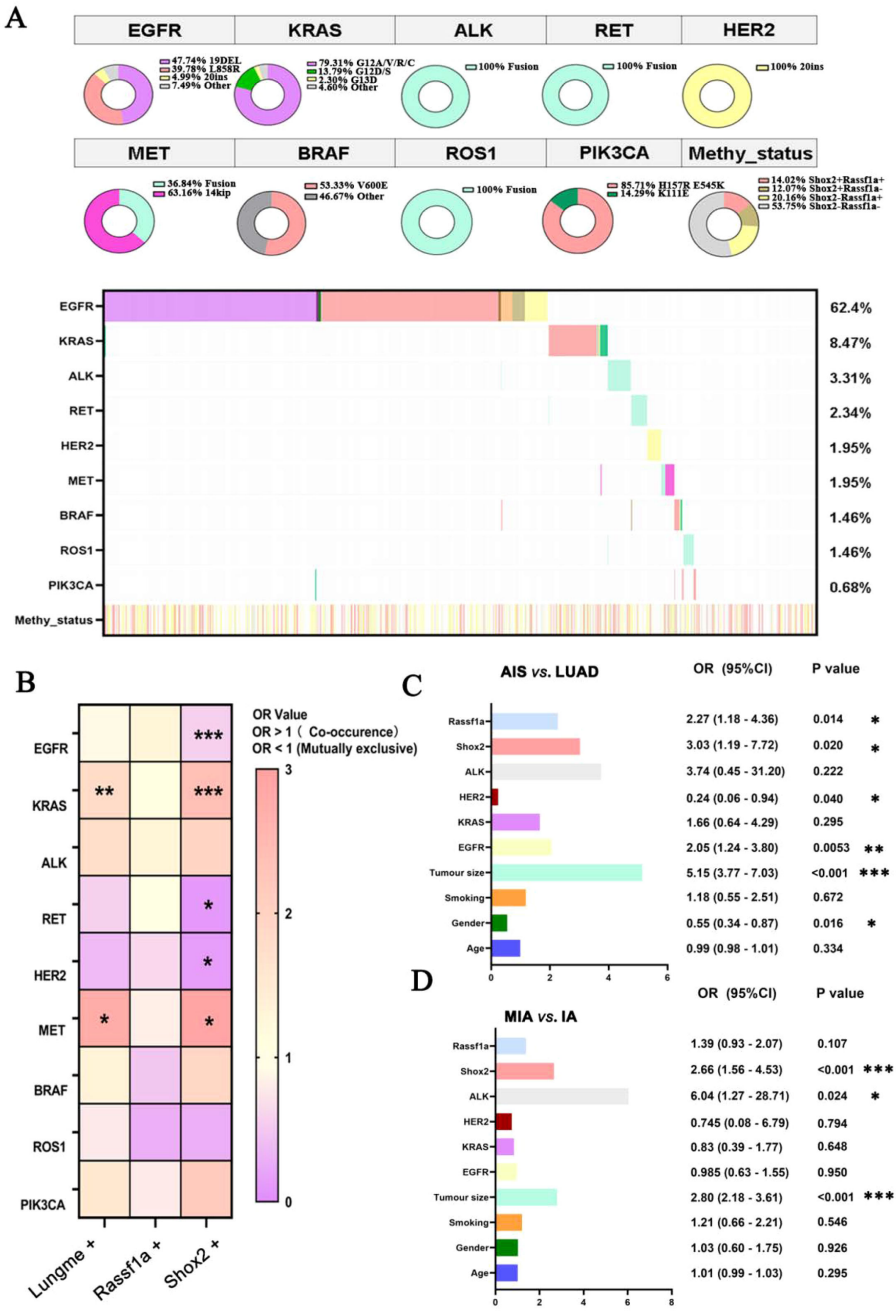
Association analysis between Shox2 and Rassf1a methylation and driver gene mutations. **(A)** Mutation landscape of 9 driver genes. **(B)** Analysis of co-occurrence and mutual exclusivity between Shox2/Rassf1a methylation and driver mutations. **(C, D)** Analysis of risk factors of the invasiveness in LUAD. *p < 0.05, **p < 0.01, ***p < 0.001.

Mutations of 10 oncogenes were detected by ARMS-PCR across the cohort of 1027 LUAD patients. NTRK gene mutations were not detected, so it was not shown in **Figure 4** and **Table 1**. Genetic mutations, including point mutations and fusion mutations, were detected in 852 patients (82.96%). The top three frequently mutated genes were EGFR (641/1027, 62.4%), KRAS (87/1027, 8.47%) and ALK (34/1027, 3.31%) (**Figure 4A**). The main mutation types of EGFR were 19DEL (306/641, 47.74%), L858R (255/641, 39.78%) and 20ins (32/641, 4.99%). Furthermore, chi-square test was used to determine the co-occurrence/mutually exclusive relationships between genetic mutations and methylation level of Shox2 and Rassf1a. Methylated Shox2 significantly co-occurred with mutations of KRAS and MET and mutually exclusive with mutations of EGFR, HER2 and RET (**Figure 4B**). Combined analysis of Shox2 and Rassf1a (Lungme assay) had a co-occurrence relationship with mutations of KRAS and MET which may be caused by methylated Shox2 (**Figure 4B**). Multiple regression analysis was used to identify stage-specific risk factors. Age, gender, tumor size, methylated Shox2, methylated Rassf1a and mutations of EGFR, KRAS, HER2 and ALK were significantly related to the invasiveness of LUAD (**Figures 4C, D**). Methylation of Shox2 and Rassf1a exerted an influence on the invasiveness of LUAD, as indicated by an odds ratio (OR) of 3.03 and 2.27 for LUAD (**Figure 4C**). For IA in stage I, the OR values of Shox2 and Rassf1a were 2.66 and 1.39 (**Figure 4D**). These results demonstrate that Shox2 methylation acts as an independent risk factor in both key comparisons: IA versus MIA and AIS versus LUAD. In contrast, Rassf1a methylation was an independent risk factor only in the AIS versus LUAD comparison. Therefore, Shox2 appears to possess superior discriminatory power for assessing the invasiveness of lung adenocarcinoma.

**Table 1:** Relationship among clinical characteristics, clinical stage, and genetic mutations of 8 genes.

Genetic mutation of 8 genes										
Variables	EGFR (n = 635)	KRAS (n = 83)	ALK (n = 33)	RET (n = 24)	HER2 (n = 20)	MET (n = 17)	BRAF (n = 11)	ROS1 (n = 13)	No mutation (n = 175)	*P* value
Mean age at diagnosis, year (Mean, IQR)	59.4 (53, 67)	62.8 (57,68.5)	57.7 (51.5, 67.5)	55.5 (48.3, 64)	48 (37.8, 54)	67.6 (61, 72.5)	60.1 (56.5, 67)	56.1 (51.5,65)	58.6 (52, 68)	<0.001
Sex										
Female	434 (68.3%)	18 (21.7%)	20 (60.6%)	16 (66.7%)	16 (80.0%)	8 (47.1%)	4 (36.4%)	11 (84.6%)	88 (50.3%)	<0.001
Male	201 (31.7%)	65 (78.3%)	13 (39.4%)	8 (33.3%)	4 (20.0%)	9 (52.9%)	7 (63.6%)	2 (15.4%)	87 (49.7%)	
Smoking										
Yes	117 (18.4%)	51 (61.4%)	6 (18.2%)	5 (20.8%)	3 (15.0%)	4 (23.5%)	5 (45.5%)	1 (7.7%)	62 (35.4%)	<0.001
No	518 (81.6%)	32 (38.6%)	27 (81.8%)	19 (79.2%)	17 (85.0%)	13 (76.5%)	6 (54.5%)	12 (92.3%)	113 (64.6%)	
Drinking										
Yes	82 (12.9%)	24 (28.9%)	4 (12.1%)	5 (20.8%)	2 (10.0%)	1 (5.9%)	2 (18.2%)	1 (7.7%)	31 (17.7%)	0.0163
No	553 (87.1%)	59 (71.1%)	29 (87.9%)	19 (79.2%)	18 (90.0%)	16 (94.1%)	9 (81.8%)	12 (92.3%)	144 (82.3%)	
Family history										
Yes	86 (13.5%)	8 (9.6%)	4 (12.1%)	3 (12.5%)	2 (10.0%)	3 (17.6%)	2 (18.2%)	3 (23.1%)	17 (9.7%)	0.799
No	549 (86.5%)	75 (90.4%)	29 (87.9%)	21 (87.5%)	18 (90.0%)	14 (82.4%)	9 (81.8%)	10 (76.9%)	158 (90.3%)	
Location of pulmonary nodules										
Left	275 (43.3%)	30 (36.1%)	18 (54.5%)	14 (58.3%)	9 (45.0%)	11 (64.7%)	7 (63.6%)	5 (38.5%)	81 (46.3%)	0.214
Right	360 (56.7%)	53 (63.9%)	15 (45.5%)	10 (41.7%)	11 (55.0%)	6 (35.3%)	4 (36.4%)	8 (61.5%)	94 (53.7%)	
Radiological features										
Solid	116 (18.3%)	26 (31.3%)	10 (30.3%)	2 (8.3%)	2 (10.0%)	2 (11.8%)	1 (9.1%)	2 (15.4%)	42 (24.0%)	<0.001
Ground-glass	114 (18.0%)	17 (20.5%)	3 (9.1%)	6 (25.0%)	12 (60.0%)	4 (23.5%)	1 (9.1%)	3 (23.1%)	39 (22.3%)	
Mix Ground-glass	275 (43.3%)	22 (26.5%)	8 (24.2%)	10 (41.7%)	2 (10.0%)	8 (47.1%)	3 (27.3%)	5 (38.5%)	63 (36.0%)	
Other	114 (17.9%)	15 (18.1%)	10 (30.3%)	5 (20.8%)	1 (5.0%)	3 (17.7%)	6 (54.6%)	3 (22.1%)	27 (15.5%)	
NA	16 (2.5%)	3 (3.6%)	2 (6.1%)	1 (4.2%)	3 (15.0%)	0 (0%)	0 (0%)	0 (0%)	4 (2.3%)	
Clinical stage										<0.001
0	32 (5.0%)	5 (6.0%)	0 (0%)	0 (0%)	9 (45.0%)	1 (5.9%)	0 (0%)	1 (7.7%)	20 (11.4%)	
I	504 (79.3%)	68 (77.9%)	27 (81.9%)	22 (91.7%)	10 (50%)	14 (82.4%)	10 (91%)	11 (84.7%)	132 (75.4%)	
II	83 (13%)	6 (7.2%)	5 (18.2%)	1 (4.2%)	1 (5.0%)	2 (11.8%)	1 (9.1%)	1 (7.7%)	19 (10.8%)	
III	10 (1.6%)	3 (3.6%)	0 (0%)	1 (4.2%)	0 (0%)	0 (0%)	0 (0%)	0 (0%)	3 (1.7%)	
IV	5 (0.8%)	1 (1.2%)	0 (0%)	0 (0%)	0 (0%)	0 (0%)	0 (0%)	0 (0%)	1 (0.6%)	
Methylation status										<0.001
Shox2+Rassf1a-	57 (9.0%)	23 (27.7%)	7 (21.2%)	0 (0%)	0 (0%)	7 (41.2%)	5 (45.4%)	3 (23.1%)	23 (13.1%)	
Shox2-Rassf1a +	151 (23.8%)	12 (14.5%)	7 (21.2%)	5 (20.8%)	4 (20%)	5 (29.4%)	1 (9.1%)	0 (0%)	18 (10.3%)	
Shox2+Rassf1a+	81 (12.8%)	13 (15.6%)	3 (9.1%)	1 (4.2%)	1 (5%)	1 (5.9%)	1 (9.1%)	1 (7.7%)	33 (18.9%)	
Shox2-Rassf1a-	346 (54.4%)	35 (52.2%)	13 (39.5%)	18 (75%)	15 (75%)	4 (23.5%)	4 (36.4%)	9 (69.2%)	101 (57.7%)	

Data are n (%); NA, not applicable or missing; IQR, interquartile range; LUAD, lung adenocarcinoma.

Meanwhile, we analyzed the relationships between genetic mutations of 8 genes and clinical characteristics. The number of patients with PIK3CA mutations was insufficient to conduct statistical analysis, therefore it was not included in **Table 1**. In addition, we excluded cases with multiple genetic mutations. The results demonstrated that different gene mutations were significantly associated with age, sex, smoking, drinking, radiological features and clinical stage (**Table 1**). Regarding radiological features, mix ground-glass nodule taken higher proportion in patients with EGFR mutation (275/635, 43.3%), RET mutation (10/24, 41.7%), and MET mutation (8/17, 47.1%) than those with other genetic mutations. However, patients with KRAS mutation (26/83, 31.3%) and ALK mutation (10/33, 30.3%) had a higher proportion of solid nodule compared with those with other mutations. For clinical stage, patients with HER2 mutation had higher proportions of stage 0 (9/20, 45%) and stage I (0/20, 50%) than those with other mutations.

### 3.5 Diagnostic utility and associations among genetic mutations, methylation profiles, and clinicopathological features​

**Figure 5:**
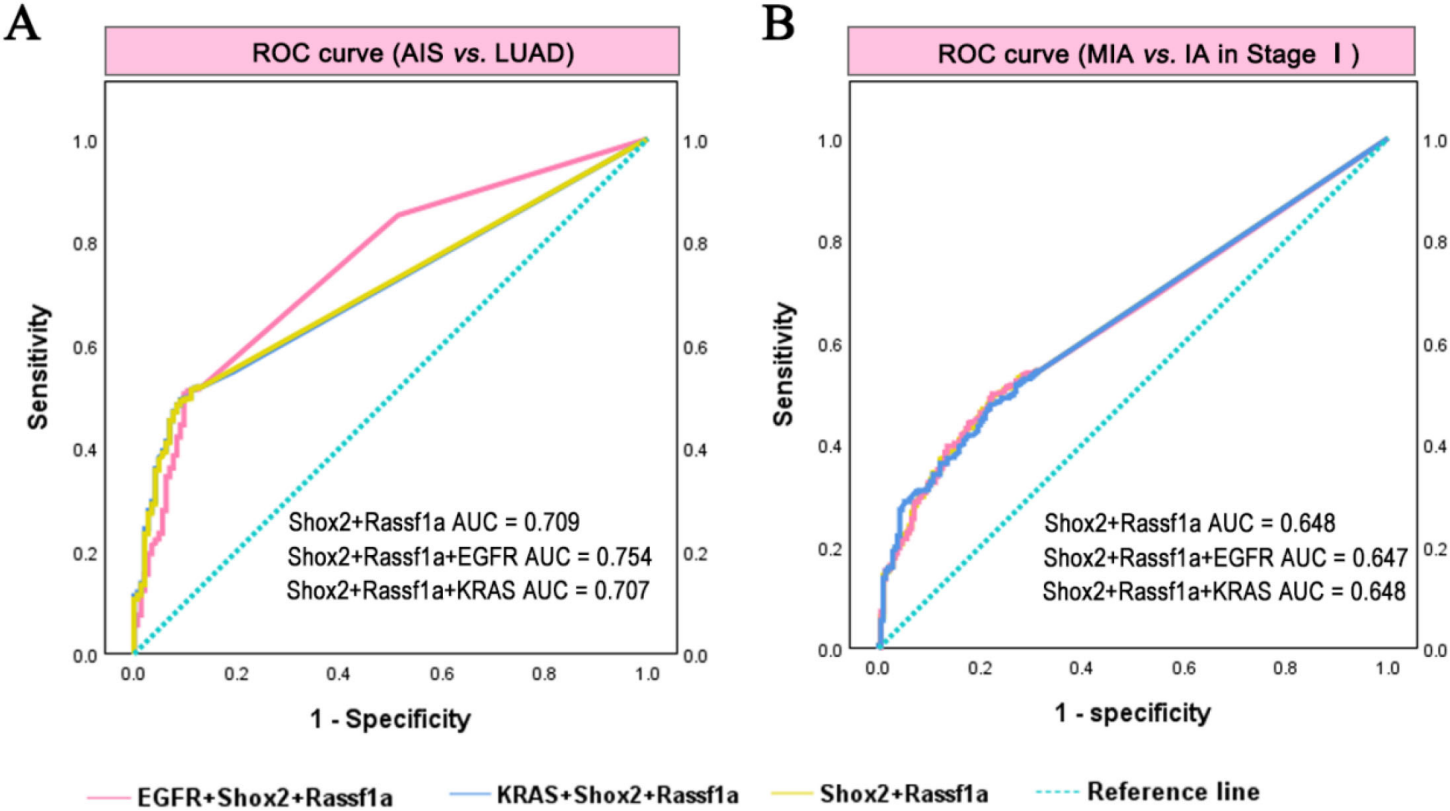
The diagnostic efficiency of combined DNA methylation and mutations of EGFR or KRAS for MIA and AIS. **(A)** ROC analysis for discriminating AIS and LUAD (stage I-IV) based on Shox2 and Rassf1a methylation combining EGFR mutations. **(B)** ROC analysis for discriminating MIA and IA in stage I based on Shox2 and Rassf1a methylation combining EGFR mutations. **(C)** ROC analysis for discriminating AIS and LUAD (stage I-IV) based on Shox2 and Rassf1a methylation combining KRAS mutations. **(E)** ROC analysis for discriminating MIA and IA in stage I based on Shox2 and Rassf1a methylation combining KRAS mutations.

EGFR and KRAS were the most frequently mutated genes in our study, and these mutations have been previously associated with the prognosis and proliferation of LUAD (11, 12). To evaluate the diagnostic efficiency of methylation markers and EGFR/KRAS mutations, ROC curve analysis was performed. For differentiating AIS from LUAD, the AUC values were 0.709 (Shox2+Rassf1a), 0.754 (Shox2+Rassf1a+EGFR), 0.707 (Shox2+Rassf1a+KRAS) (**Figure 5A**). The sensitivity of Shox2+Rassf1a, Shox2+Rassf1a+EGFR, Shox2+Rassf1a+KRAS was 51.3%, 50.5%, and 49.4% with specificity of 88.9%, 89.6%, and 91%, respectively. For differentiating MIA from IA in stage I, the corresponding AUCs were 0.648, 0.647, and 0.648 (**Figure 5B**). The sensitivity of Shox2+Rassf1a, Shox2+Rassf1a+EGFR, Shox2+Rassf1a+KRAS was 49.5%, 49.9%, and 47.7% with specificity of 76.7%, 77.7%, and 68.4%, respectively. These results indicated that combining EGFR mutations could improve the diagnostic efficiency of early-stage lesions (AIS from LUAD across stages I-IV), but not histological progression (MIA from IA). However, KRAS mutations failed to enhance the diagnostic efficiency.

We further analyzed the differences in the methylation status among different mutation types of EGFR and KRAS (**Table 2**). The results indicated that Shox2 and Rassf1a methylation profiles of EGFR L858R and 19DEL mutations were similar, with a significantly higher proportion of Rassf1a methylation compared to the 20ins and other mutation types. In contrast, the 20ins and other mutation subtypes exhibited a higher proportion of double-negative methylation status (Shox2-/Rassf1a-) than L858R and 19DEL. These findings suggested that EGFR L858R and 19DEL mutations may be more prone to methylation alterations, particularly Rassf1a methylation, compared to the 20ins mutation. In contrast, no significant differences in methylation patterns were observed among different KRAS mutation subtypes.

**Table 2:** Association between EGFR and KRAS mutation subtypes and promoter methylation status of Shox2 and Rassf1a.

Genetic mutation of EGFR and KRAS										
Variables	EGFR (n = 635)					P value	KRAS (n = 83)			*P* value
	L858R (n = 302)		19DEL (n = 253)	20ins (n = 32)	Other (n = 48)		G12A/V/R/C (n = 66)	G12D/S (n = 11)	Other (n = 6)	
Methylation status										
Shox2+Rassf1a-		26 (8.6%)	22 (8.7%)	5 (15.6%)	3 (6.3%)	<0.001	17 (25.8%)	3 (27.3%)	1 (16.7%)	0.51
Shox2-Rassf1a +		82 (27.2%)	58 (22.9%)	4 (12.5%)	7 (14.6%)		9 (13.6%)	1 (9.1%)	1 (16.7%)	
Shox2+Rassf1a+		42 (13.9%)	29 (11.4%)	3 (9.4%)	7 (14.6%)		12 (18.2%)	1 (9.1%)	3 (50%)	
Shox2-Rassf1a-		152 (50.3%)	144 (56.9%)	20 (62.5%)	31 (64.5%)		28 (42.4%)	6 (54.5%)	1 (16.7%)	

Data are n (%).

As demonstrated in **Tables 3** and **4**, distinct methylation statuses remained significantly associated with clinical stage across different genetic mutation subtypes. Patients in the double-negative group (Group 4) exhibited a significantly higher proportion of AIS compared to methylation-positive groups (Group 1-3). In contrast, the double-positive group (Group 3) showed a higher percentage of patients with stage III–IV LUAD. Furthermore, significant differences in methylation patterns between minimally MIA and IA within stage I were observed only in EGFR L858R and 19DEL mutations, but not in KRAS mutations. This lack of association in KRAS-mutant cases may be attributed to the inherently more aggressive nature of KRAS-driven tumors and small sample size. The associations between methylation-based subgroups and clinical characteristics within each mutation subtype were investigated (**Supplementary Tables S2**, **S3**). Our analysis revealed that patients with the EGFR L858R mutation in Group 1 (Shox2+Rassf1a-) were significantly older than those in other subgroups. In contrast, no significant differences in age distribution were observed across methylation subgroups for either the EGFR 19DEL or KRAS mutations. Furthermore, methylation status was significantly associated with smoking history and alcohol consumption in both KRAS and EGFR 19DEL cohorts. A significant correlation with gender was only identified across methylation subgroups in cases harboring EGFR 19DEL mutation.

**Table 3:** Association between EGFR L858R and 19DEL mutation subgroups, defined by Shox2 and Rassf1a methylation status, and clinical stage and pathological grade in stage I.

Genetic mutation of EGFR										
Variables	L858R				P value	19DEL				*P* value
	Group 1 (n =26)	Group 2 (n = 82)	Group 3 (n = 42)	Group 4 (n = 152)		Group 1 (n = 22)	Group 2 (n = 58)	Group 3 (n = 29)	Group 4 (n = 144)	
Clinical stage										
0	1 (3.8%)	5 (6.1%)	1 (2.4%)	19 (12.5%)	<0.001	0 (0%)	3(5.2%)	1(3.5%)	27 (18.8%)	<0.001
I	23 (88.6%)	67 (81.8%)	20 (47.6%)	120 (78.9%)		17 (77.3%)	43 (74.1%)	17 (58.6%)	101 (70.1%)	
II	1 (3.8%)	6 (7.3%)	10 (23.8%)	5 (3.3%)		3 (13.5%)	5 (8.6%)	3 (10.3%)	11 (7.6%)	
III	1 (3.8%)	3 (3.7%)	10 (23.8%)	5 (3.3%)		1 (4.6%)	5 (8.6%)	6 (20.7%)	2 (1.4%)	
IV	0 (0%)	1 (1.2%)	1 (2.4%)	3 (2.0%)		1 (4.6%)	2 (3.5%)	2 (6.9%)	3 (2.1%)	
Pathological grade*										
MIA	3 (13.0%)	22 (32.8%)	3 (15%)	42 (35%)	0.046	1 (5.9%)	10 (23.3%)	1 (5.9%)	41 (40.6%)	<0.001
IA	20 (87.0%)	45 (67.2%)	17 (85%)	78 (65%)		16 (94.1%)	33 (76.7%)	16 (94.1%)	60 (59.4%)	

Data are n (%); *only patients in stage I were included in pathological grade analysis; Group 1: Shox2+Rassf1a-, Group 2: Shox2-Rassf1a+, Group 3: Shox2+Rassf1a+, Group 4: Shox2-Rassf1a; MIA, minimally invasive adenocarcinoma; IA, invasive adenocarcinoma.

**Table 4:** Association between KRAS G12A/V/R/C mutation subgroup, defined by Shox2 and Rassf1a methylation status, and clinical stage and pathological grade in stage I.

Genetic mutation of KRAS					
Variables	G12A/V/R/C				
	Group 1 (n = 17)	Group 2 (n = 9)	Group 3 (n = 12)	Group 4 (n = 28)	P value
Clinical stage					
0	0 (0%)	0(0%)	0 (0%)	7 (25.0%)	0.007
I	10 (58.8%)	8 (88.9%)	10 (83.4%)	18 (64.2%)	
II	3 (17.7%)	0 (0%)	1 (8.3%)	1 (3.6%)	
III	4 (23.5%)	1 (11.1%)	1 (8.3%)	1 (3.6%)	
IV	0 (0%)	0 (0%)	0 (0%)	1 (3.6%)	
Pathological grade*					
MIA	5 (50%)	1 (12.5%)	1 (10%)	5 (27.8%)	0.105
IA	5 (50%)	7 (87.5%)	9 (90%)	13 (72.8%)	

Data are n (%); *only patients in stage I were included in pathological grade analysis; Group 1: Shox2+Rassf1a-, Group 2: Shox2-Rassf1a+, Group 3: Shox2+Rassf1a+, Group 4: Shox2-Rassf1a; MIA, micro invasive adenocarcinoma; IA, invasive adenocarcinoma.

## 4 Discussion

Lung cancer ranks as the second most prevalent malignancy, with an 11.4% incidence rate. Non-small cell lung cancer (NSCLC) is the most common form of lung cancer, accounting for over 80% of all cases. LUAD, the predominant histologic subtype, accounts for approximately 45% of all NSCLC diagnoses (13). Currently, Shox2 and Rassf1a methylation testing is utilized for the early detection and diagnosis of lung cancer in clinical practice, while molecular profiling of tumor tissue for genetic mutations is widely employed to guide targeted therapy selection. Despite significant progress in these molecular diagnostics over the last ten years, their full clinical significance requires further investigation. Emerging evidence indicates that specific DNA methylation signatures and driver mutations may represent specific lung cancer subsets with divergent prognoses (14, 15), potentially facilitating personalized therapeutic strategies. However, these findings are limited by sample size, geographic origin, and ethnic diversity. Validation through more clinical studies remains necessary. Therefore, this study enrolled 1027 LUAD patients across various clinical stages and histological subtypes. Clinical records, along with genetic and methylation testing results, were collected. We analyzed associations between tissue Shox2 and Rassf1a methylation and clinical features/disease malignancy. Furthermore, we evaluated the discriminatory power of combining methylation markers with gene mutation status for identifying disease malignancy.

We found that tissue Shox2 and Rassf1a methylation were associated with sex, age, smoking, drinking, and TNM stage and IA exhibited significantly higher methylation levels than MIA which were consistent with previous reports (16). Given that chronic smoking and drinking affect methylation and are independent lung cancer risk factors, future work should elucidate whether their carcinogenic and prognostic influences are mediated by epigenetic changes. Since Shox2 and Rassf1a methylation correlate with TNM stage and tumor grade, we assessed whether Shox2 and Rassf1a methylation could stratify malignant potential. As diagnostic biomarkers for lung cancer, detection of Shox2 and Rassf1a methylation achieved sensitivity ranging from 71.5% to 97% and specificity between 82.3% and 100% (17). However, in our study, the sensitivity for LUAD (stage I-IV) was 48.38%. The relatively low overall sensitivity may stem from cohort bias due to the predominance of stage I patients (70.3%, 722/1027). Sensitivity for stages II-III remained consistent with prior reports. Comparative analysis of Shox2 and Rassf1a methylation positivity rates revealed significantly lower levels in AIS and stage I versus stages II-III, suggesting a positive correlation between methylation burden and disease malignancy. Notably, the 50% sensitivity in stage IV cases should be interpreted with caution given the limited sample size (1.75%, 18/1027). Further optimization of the cut-off value contributed to enhanced discriminatory performance of the Lungme assay in distinguishing LUAD from AIS or IA from MIA. ROC analysis demonstrated that combined detection of Shox2 and Rassf1a methylation provided significantly superior discriminatory power for distinguishing AIS from LUAD (stage I-IV) (AUC = 0.709) and MIA from IA (AUC = 0.648) compared to either biomarker alone (AUC values between 0.5 and 0.6), which suggested that Shox2 or Rassf1a methylation alone demonstrates suboptimal discriminatory efficacy. Future research may need to incorporate additional methylation markers for a more robust pathological grading analysis. A prior small-scale study (n=258) reported AUC values of 0.653 (Shox2) and 0.664 (Rassf1a) for distinguishing AIS from LUAD (stage I-IV), but did not evaluate the efficiency of Shox2 combined Rassf1a (16). The AUC differences may reflect variations in statistical power due to disparate sample sizes. Compared to our methylation markers, imaging-based models from previous research show superior performance in discriminating lung adenocarcinoma invasiveness, with reported sensitivity exceeding 80% and AUC values above 0.8 (18, 19). Nevertheless, the diagnostic performance of methylation markers alone remains limited. When combined with radiological descriptions, their ability to differentiate between AIS and LUAD was augmented, indicating that these modalities play complementary roles. Therefore, future clinical research should focus on developing an integrated model incorporating multiple parameters to guide decision-making.

Detection of driver mutations not only guides the selection of targeted therapeutic strategies but also carries significant prognostic implications. We compiled the driver mutation results from all cases, generating a comprehensive driver mutation landscape. EGFR (62.4%) and KRAS (8.47%) emerged as the most frequently altered genes. Cumulative alterations in DNA promoter methylation may contribute to oncogenesis by predisposing to DNA mutations (20–22). Therefore, we investigated the patterns of co-occurrence and mutual exclusivity between DNA methylation events and driver mutations. Significant co-occurrence was observed between Shox2 methylation and mutations in MET and KRAS, while it exhibited mutual exclusivity with mutations in EGFR, RET, and HER2. The functional proteins encoded by EGFR, RET, and HER2 all belong to the receptor tyrosine kinase family. The mutual exclusivity with Shox2 methylation which strongly suggests the existence of distinct LUAD molecular subtypes characterized by divergent molecular features and oncogenic pathways. While KRAS mutation is reportedly mutually exclusive with EGFR mutation and is associated with enhanced tumor aggressiveness in LUAD (23, 24), the observed co-occurrence of Shox2 methylation with KRAS mutation raises the possibility of cooperative potentiation of invasive potential during LUAD pathogenesis. Consequently, this finding prompts a critical question: Can Shox2 methylation status enable refined prognostic stratification of LUAD patients based on their specific mutational profiles? Definitive assessment of this potential requires longitudinal clinical tracking of patient outcomes. Unlike Shox2, no significant associations (either co-occurrence or mutual exclusivity) were detected between Rassf1a methylation and these driver gene mutations. This intriguing finding suggests that Rassf1a methylation may represent a more ubiquitous or fundamental early event in LUAD pathogenesis, occurring independently of these driver mutations. Alternatively, it is indicated that Rassf1a may promote tumorigenesis through distinct mechanisms or function effectively across diverse molecular contexts. These distinct association patterns underscore the complexity of LUAD’s molecular pathogenesis, highlighting key knowledge gaps requiring further mechanistic investigation. Given the established prognostic potential of driver mutations, we further evaluated the discriminatory capacity of combined Shox2 and Rassf1a methylation and EGFR/KRAS mutations to stratify LUAD by malignant potential. Notably, the inclusion of EGFR or KRAS mutation enhanced the discriminatory efficiency for distinguishing AIS from LUAD. This demonstrates a synergistic effect, indicating that integrating driver mutation testing with methylation assay can refine diagnostic precision and facilitates the stratified management of LUAD patients.

Our further findings demonstrate that distinct methylation patterns of Shox2 and Rassf1a are associated with different genetic mutations of EGFR and KRAS and are significantly correlated with clinical stage in LUAD. Moreover, the clinical features linked to specific methylation statuses varied across EGFR and KRAS mutation subtypes. These observations suggest that the combined Shox2 and Rassf1a methylation, together with genetic mutation profiles, may facilitate the stratification of LUAD patients into more refined subtypes. Such molecular subtyping could support improved patient stratification and personalized clinical management, thereby advancing precision medicine in LUAD. This study can serve as a supplement to traditional pathology in diagnostically challenging situations, such as when small biopsy specimens yield unclear results or when intraoperative frozen section diagnosis is difficult. Our subsequent research will focus on exploring these specific clinical pathways where its advantages can be leveraged and promoting the advancement of more accessible detection technologies. The ultimate goal is to leverage existing test results in clinical practice to enable a more holistic assessment of patient condition.

However, several limitations warrant consideration: 1) The retrospective design establishes observational associations between Shox2 and Rassf1a methylation and clinical features but cannot establish causality. 2) The mechanistic basis underlying the observed co-occurrence/mutual exclusivity of Shox2 methylation with driver mutations remains unclear. 3) The absence of patient survival data precludes direct prognostic evaluation of these markers. Future studies must incorporate longitudinal survival analysis to directly evaluate the prognostic value of methylation markers and elucidate the mechanistic interplay between Shox2 methylation and driver mutations to advance our understanding of LUAD pathogenesis and therapeutic development.

## 5 Conclusions

This study analyzed clinical data, imaging, histopathology, Shox2 and Rassf1a methylation, and driver mutation status in 1027 LUAD patients and explored the integrated diagnostic strategy. We found that Shox2 and Rassf1a methylation correlated with age, sex, smoking, alcohol and driver mutations. The combined analysis of Shox2 and Rassf1a methylation demonstrates superior diagnostic accuracy for differentiating AIS from LUAD (stage I-IV) and MIA from IA compared to individual markers. Integrating these methylation markers with driver gene mutation status provide a modest improvement in discriminatory efficacy. Therefore, these clinical molecular tests, used for diagnosis and targeted therapy selection, also offer valuable guidance for patient prognostic stratification.

## Data Availability

The original contributions presented in the study are included in the article/**Supplementary Material**. Further inquiries can be directed to the corresponding author.
