# Supplementary material for: Shox2 and Rassf1a DNA methylation: diagnostic utility and association with clinical stage, histological progression and gene mutational landscape in lung adenocarcinoma
Source: Front Oncol. 2026 Jan 28;16:1727695. doi: 10.3389/fonc.2026.1727695 (PMC12890684; doi:10.3389/fonc.2026.1727695)
Supplement: Supplementary file 1 [file Table1.docx]

Supplementary Material

# Supplementary Tables

**Supplementary Table 1.** Relationships among baseline characteristics, Shox2 and Rassf1a methylation and clinical stage in LUAD patients.

|  |  | **Clinical stage** | | | | |  |
| --- | --- | --- | --- | --- | --- | --- | --- |
| **Variables** | **Overall**  **(n = 1027)** | **0 (n = 144)** | **Ⅰ (n = 722)** | **Ⅱ (n = 78)** | **Ⅲ (n = 65)** | **Ⅳ (n = 18)** | ***P* value** |
| **Mean age at diagnosis, year (Mean, IQR)** | **59**  **(53, 67)** | **53**  **(46, 69.5)** | **60**  **(54, 68)** | **61**  **(54, 69)** | **61**  **(54, 69)** | **58**  **(56, 64)** | **0.587** |
| **Sex** |  |  |  |  |  |  |  |
| **Female** | **625 (60.85%)** | **107 (74.30%)** | **429 (59.42%)** | **41 (52.56%)** | **34 (52.31%)** | **14 (77.78%)** | **0.001** |
| **Male** | **402 (39.14%)** | **37 (25.70%)** | **293 (40.58%)** | **37 (47.44%)** | **31 (47.59%)** | **4 (22.22%)** |  |
| **Smoking** |  |  |  |  |  |  |  |
| **Yes** | **257 (25.02%)** | **17 (11.81%)** | **194 (26.87%)** | **23 (29.49%)** | **20 (30.77%)** | **3 (16.67%)** | **0.002** |
| **No** | **770 (74.98%)** | **127 (88.19%)** | **528 (73.13%)** | **55 (70.51%)** | **45 (69.23%)** | **15 (83.33%)** |  |
| **Drinking** |  |  |  |  |  |  |  |
| **Yes** | **154 (15%)** | **25 (17.36%)** | **116 (16.07%)** | **13 (16.67%)** | **8 (12.30%)** | **1 (5.56%)** | **0.386** |
| **No** | **873 (85%)** | **119 (82.64%)** | **606 (83.93%)** | **65 (83.33%)** | **57 (87.69%)** | **17 (94.44%)** |  |
| **Family history** |  |  |  |  |  |  |  |
| **Yes** | **129 (12.56%)** | **25 (17.36%)** | **91 (12.60%)** | **3 (3.85%)** | **7 (10.77%)** | **3 (16.67%)** | **0.064** |
| **No** | **898 (87.44%)** | **119 (82.64%)** | **631 (87.40%)** | **75 (96.15%)** | **58 (89.23%)** | **15 (83.33%)** |  |
| **Location of pulmonary nodules-** |  |  |  |  |  |  |  |
| **left** | **458 (44.60%)** | **67 (46.53%)** | **318 (44.04%)** | **33 (42.31%)** | **30 (46.15%)** | **10 (55.56%)** | **0.842** |
| **Right** | **569 (55.40%)** | **77 (53.47%)** | **404 (55.96%)** | **45 (57.69%)** | **35 (53.85%)** | **8 (44.44%)** |  |
| **Radiological features** |  |  |  |  |  |  |  |
| **Solid** | **206 (20.06%)** | **6 (4.17%)** | **134 (18.56%)** | **31 (39.74%)** | **29 (44.62%)** | **6 (33.33%)** | **＜0.001** |
| **Ground-glass** | **200 (19.47%)** | **78 (54.16%)** | **118 (16.34%)** | **2 (2.56%)** | **2 (3.08%)** | **0 (0%)** |  |
| **Mix Ground-glass** | **401 (39.05%)** | **41 (28.47%)** | **339 (46.95%)** | **14 (17.95%)** | **6 (4.62%)** | **1 (5.56%)** |  |
| **Other** | **190 (18.50%)** | **4 (2.78%)** | **122 (16.90%)** | **28 (35.90%)** | **27 (41.54%)** | **9 (50%)** |  |
| **NA** | **30 (2.92%)** | **15 (10.42%)** | **9 (1.25%)** | **3 (3.85%)** | **1 (1.54%)** | **2 (11.11%)** |  |
| **Methylation status** |  |  |  |  |  |  |  |
| **Shox2+Rassf1a-** | **124 (12.07%)** | **3 (2.08%)** | **92 (12.74%)** | **15 (19.23%)** | **11 (16.92%)** | **3 (16.67%)** | **＜0.001** |
| **Shox2-Rassf1a +** | **207 (20.16%)** | **13 (9.03%)** | **164 (22.71%)** | **15 (19.23%)** | **12 (18.46%)** | **3 (16.67%)** |  |
| **Shox2+Rassf1a+** | **144 (14.02%)** | **3 (2.08%)** | **87 (12.05%)** | **26 (33.33%)** | **25 (38.46%)** | **3 (16.67%)** |  |
| **Shox2-Rassf1a-** | **552 (53.75%)** | **125 (86.81%)** | **379 (52.49%)** | **22 (28.21%)** | **17 (26.15%)** | **9 (50%)** |  |

Note: Data are n (%); NA=not applicable or missing; IQR: interquartile range; LUAD: lung adenocarcinoma.

**Supplementary Table 2.** Association between EGFR L858R and 19DEL mutation subgroups, defined by Shox2 and Rassf1a methylation status, and clinical characteristics.

|  | **Genetic mutation of EGFR** | | | | | | | | | |
| --- | --- | --- | --- | --- | --- | --- | --- | --- | --- | --- |
| **Variables** | **L858R** | | | | **19DEL** | | | |  |  |
|  | **Group 1**  **(n =26)** | **Group2**  **(n = 82)** | **Group3**  **(n = 42)** | **Group4**  **(n = 152)** | **P value** | **Group 1**  **(n = 22)** | **Group2**  **(n = 58)** | **Group3**  **(n = 29)** | **Group4**  **(n = 144)** | ***P* value** |
| **Mean age at diagnosis, year (Mean, IQR)** | **64.7**  **(60, 71)** | **61.3**  **(55, 68)** | **61.5**  **(54.5, 67.5)** | **59.5**  **(52, 67)** | **0.027** | **59.1**  **(53, 64)** | **59.2**  **(52, 69.8)** | **57.6**  **(54, 62)** | **57.1**  **(52, 65)** | **0.717** |
| **Sex** |  |  |  |  |  |  |  |  |  |  |
| **Female** | **16 (61.5%)** | **56 (68.3%)** | **27 (64.3%)** | **115 (75.7%)** | **0.269** | **15 (68.2%)** | **35 (60.3%)** | **11(37.9%)** | **107 (74.3%)** | **<0.001** |
| **Male** | **10 (38.5%)** | **26 (31.7%)** | **15 (35.7%)** | **37 (24.3%)** |  | **7 (31.8%)** | **23 (39.7%)** | **18(62.1%)** | **37 (25.7%)** |  |
| **Smoking** |  |  |  |  |  |  |  |  |  |  |
| **Yes** | **6(23.1%)** | **14(17.1%)** | **12(28.6%)** | **18(11.8%)** | **0.051** | **17(77.3%)** | **9(15.5%)** | **9 (31.0%)** | **16(11.1%)** | **<0.001** |
| **No** | **20 (76.9%)** | **68 (82.9%)** | **30 (71.4%)** | **135 (88.2%)** |  | **5 (22.7%)** | **49 (84.5%)** | **20(69.0%)** | **128 (88.9%)** |  |
| **Drinking** |  |  |  |  |  |  |  |  |  |  |
| **Yes** | **4 (15.4%)** | **13 (84.2%)** | **7 (16.7%)** | **11 (7.2%)** | **0.128** | **5 (22.7%)** | **7 (12.1%)** | **7 (24.1%)** | **12 (8.3%)** | **0.043** |
| **No** | **22 (84.6%)** | **69 (15.8%)** | **35 (83.3%)** | **141 (8%)** |  | **17 (77.3%)** | **51 (87.9%)** | **22(75.9%)** | **132 (91.7%)** |  |
| **Family history** |  |  |  |  |  |  |  |  |  |  |
| **Yes** | **3 (11.5%)** | **10 (12.2%)** | **7 (16.7%)** | **21 (13.8%)** | **0.902** | **5 (22.7%)** | **10 (17.2%)** | **5 (17.2%)** | **17 (11.8%)** | **0.460** |
| **No** | **23 (88.5%)** | **72 (87.8%)** | **35 (83.3%)** | **131 (86.2%)** |  | **17 (77.3%)** | **48 (82.8%)** | **24(82.8%)** | **127 (88.2%)** |  |
| **Location of pulmonary nodules-** |  |  |  |  |  |  |  |  |  |  |
| **left** | **12 (46.2%)** | **32 (39.0%)** | **18 (42.9%)** | **61 (40.1%)** | **0.915** | **13 (59.1%)** | **27 (46.6%)** | **13(44.8%)** | **64 (54.4%)** | **0.640** |
| **Right** | **14 (53.8%)** | **50 (61.0%)** | **24 (57.1%)** | **91 (59.9%)** |  | **9 (40.9%)** | **31 (53.4%)** | **16(55.2%)** | **80 (55.6%)** |  |

Note: Data are n (%); Group 1: Shox2+Rassf1a-, Group 2: Shox2-Rassf1a+, Group 3: Shox2+Rassf1a+, Group 4: Shox2-Rassf1a.

**Supplementary Table 3.** Association between KRAS G12A/V/R/C mutation subgroup, defined by Shox2 and Rassf1a methylation status, and clinical characteristics.

|  | **Genetic mutation of KRAS** | | | | |
| --- | --- | --- | --- | --- | --- |
| **Variables** | **G12A/V/R/C** | | | | |
|  | **Group 1**  **(n = 17)** | **Group2**  **(n = 9)** | **Group3**  **(n = 12)** | **Group4**  **(n = 28)** | ***P* value** |
| **Mean age at diagnosis, year (Mean, IQR)** | **66.1**  **(62, 71)** | **62.1**  **(55.7, 65.3)** | **63.2**  **(57, 68.5)** | **62.5**  **(57.5, 68)** | **0.451** |
| **Sex** |  |  |  |  |  |
| **Female** | **1 (5.9%)** | **2 (22.2%)** | **1 (8.3%)** | **9 (32.1%)** | **0.099** |
| **Male** | **16 (94.1%)** | **7 (77.8%)** | **11 (91.7%)** | **19 (67.9%)** |  |
| **Smoking** |  |  |  |  |  |
| **Yes** | **13 (76.5%)** | **7 (77.8%)** | **10 (83.3%)** | **14 (50%)** | **0.047** |
| **No** | **4 (23.5%)** | **2 (22.2%)** | **2 (16.7%)** | **14 (50%)** |  |
| **Drinking** |  |  |  |  |  |
| **Yes** | **11 (64.7%)** | **1 (11.1%)** | **3 (25%)** | **8 (28.6%)** | **0.007** |
| **No** | **6 (35.3%)** | **8 (88.9%)** | **9 (75%)** | **20 (71.4%)** |  |
| **Family history** |  |  |  |  |  |
| **Yes** | **2 (11.8%)** | **1 (11.1%)** | **0 (0%)** | **3 (10.7%)** | **0.832** |
| **No** | **15 (88.2%)** | **8 (88.9%)** | **12 (100%)** | **25 (89.3%)** |  |
| **Location of pulmonary nodules-** |  |  |  |  |  |
| **left** | **4 (23.5%)** | **3 (33.3%)** | **5 (41.7%)** | **10 (35.7%)** | **0.831** |
| **Right** | **13 (76.5%)** | **6 (66.7%)** | **7 (58.3%)** | **18 (64.3%)** |  |

Note: Data are n (%); Group 1: Shox2+Rassf1a-, Group 2: Shox2-Rassf1a+, Group 3: Shox2+Rassf1a+, Group 4: Shox2-Rassf1a.
